# Covid-19 pandemic and Brazilian Nursing: unveiling meanings of work

**DOI:** 10.1590/1980-220X-REEUSP-2022-0156en

**Published:** 2022-09-19

**Authors:** Jorge Domingos de Sousa, Kayo Henrique Jardel Feitosa Sousa, Ítalo Rodolfo Silva, Regina Célia Gollner Zeitoune

**Affiliations:** 1 Universidade Federal do Rio de Janeiro, Escola de Enfermagem Anna Nery, Rio de Janeiro, RJ, Brazil.; 2 Universidade Federal de Rondônia, Departamento de Enfermagem, Porto Velho, RO, Brazil.; 3 Universidade Federal do Rio de Janeiro, Instituto de Enfermagem, Macaé, RJ, Brazil.

**Keywords:** Nursing, COVID-19, Working Conditions, Professional Practice, Occupational Health, Enfermería, COVID-19, Condiciones de Trabajo, Práctica Profesional, Salud Laboral, Enfermagem, COVID-19, Condições de Trabalho, Prática Profissional, Saúde do Trabalhador

## Abstract

The objective of this study was to reflect on the meanings of the work of Brazilian nursing care in the context of the Covid-19 pandemic. This is a theoretical study anchored in the definition of meanings of work, according to Estelle Morin's perspective. The work developed by nursing professionals became even more evident in pandemic times, with the precarious conditions of health services in Brazil coming to light. During the pandemic, the incorporation of meanings of work became more important, given that the society recognized the relevance of these professionals in dealing with the pandemic, and this allowed the discussion about their social, political, and economic recognition. The impacts of nursing performance during the Covid-19 pandemic are related to the economic issue, social values, autonomy in the exercise of the profession, recognition, and safety, reflecting on the sense of purpose of work. Thus, the work that makes sense for nursing professionals is related to professional appreciation, specifically, to salary recognition, while what makes no sense is what hinders intellectual, cognitive, and financial progress. Thus, conditions were imposed that give directions to ambivalent meanings to work.

## RESUMO

Objetivou-se refletir sobre os sentidos do trabalho da Enfermagem assistencial brasileira no contexto da pandemia da Covid-19. Estudo teórico ancorado na definição de sentidos do trabalho, segundo a perspectiva de Estelle Morin. O trabalho desenvolvido pelos profissionais de Enfermagem ficou ainda mais evidenciado em tempos pandêmicos, vindo à tona as condições precárias dos serviços de saúde no Brasil. Durante a pandemia, a incorporação de sentidos ao trabalho passou a ter importância maior, tendo em vista que a sociedade reconheceu a relevância desses profissionais no enfrentamento da pandemia, e isso possibilitou a discussão acerca do seu reconhecimento social, político e econômico. Os impactos da atuação da Enfermagem durante a pandemia de Covid-19 têm relação com a questão econômica, valores sociais, autonomia no exercício da profissão, reconhecimento e segurança, refletindo na razão de ser do trabalho. Assim, o trabalho que faz sentido para os profissionais de Enfermagem relaciona-se à valorização profissional, especificamente, ao reconhecimento salarial, enquanto o que não faz sentido é aquele que impossibilita avanços do ponto de vista intelectual, cognitivo e financeiro. Dessarte, foram impostas condições que dão rumos para sentidos ambivalentes ao trabalho.

## RESUMEN

Se objetivó reflexionar sobre los sentidos del trabajo de la Enfermería asistencial brasileña en el contexto de la pandemia de Covid-19. Estudio teórico ancorado en la definición de sentidos del trabajo, según la perspectiva de Estelle Morin. El trabajo desarrolladlo por los profesionales de Enfermería se mostró aún más evidente em tiempos pandémicos, una vez que se puso de relieve las condiciones precarias de los servicios de salud en Brasil. Durante la pandemia, la incorporación de sentidos al trabajo pasó a tener más importancia, una vez que la sociedad reconoció la relevancia de esos profesionales en el enfrentamiento de la pandemia, y eso posibilitó la discusión acerca de su reconocimiento social, político y económico. Los impactos de la actuación de la Enfermería durante la pandemia de Covid-19 están relacionados a la cuestión económica, a los valores sociales, a la autonomía en el ejercicio de la profesión, al reconocimiento y a la seguridad, reflexionando acerca de la existencia del trabajo. Así, lo que tiene sentido para los profesionales de Enfermería se relaciona a la valorización profesional, específicamente, al reconocimiento salarial, mientras que lo que no tiene sentido es justo lo que pueda impedir avances intelectuales, cognitivos y financieros. De esa manera, se determinaron condiciones que dan rumbos para sentidos ambivalentes al trabajo.

## INTRODUCTION

Just before the dawn of 2020, a public health issue was announced to the world from Wuhan, China. Initially, without the perspective that this problem could reach global projection, people far from that province followed their lives dynamics. In effect, it was apparently just a tiny part of a complex whole of humanity, affected by yet another virus.

The problem, signaled at a specific point in the world, was quickly identified in its biological dimension, under a specific taxonomy, resulting in the name SARS-CoV-2 - popularly known as the new coronavirus –, capable of causing Covid-19, a disease that can progress to serious cases of Severe Acute Respiratory Syndrome (SARS)^(1)^. However, the world did not expect that in the first months of 2020 that public health problem in the Chinese province would result in a severe and profound practical experience wrapped in the complexity of the structural systems of humanity, with due emphasis on health systems.

The contemporary world then began to experience the pandemic of the century. With it, even if unintentionally for part of the human beings, there was the opportunity to understand that all local communities are connected to a single global social fabric. This finding indicates that a virus, when affecting small communities, can generate global impacts, at an inestimable speed, on pathological thinking. This infectious agent isolates the biological, cultural, economic and political dimensions, whose fragmentation brings as its main outcome a sharp crisis for existing social, individual, and programmatic vulnerabilities^(2)^.

In addition, the heterogeneity of the factors that make up and affect each community on the globe reveals – from the Covid-19 pandemic on – the importance of conceiving this phenomenon not only in its global projection, but also in its unique dimensions for the strategic confrontation of a disease that, until the beginning of July 2022, resulted in more than 6 million deaths worldwide, with about 650 thousand in Brazil alone^(3)^.

In this projection from the whole to the parts and from the parts to the whole – perfectly contemplated by the hologrammatic principle of complexity^(4)^ –, there is also the fact that health systems should be thought based on the human resources that constitute them. Among these professionals, there are those who represent more than half of the workers inserted in the health systems: nursing professionals. With almost 28 million workers across the planet, they form the main army on the front lines of the fight against the current pandemic^(5)^.

Thus, when conceiving the Covid-19 pandemic as a complex phenomenon, this same complexity demands important strategies that allow not only knowing this phenomenon in its multidimensionality, but, especially, promoting possibilities to, based on this discernment, advance in the actions required to face the challenges posed by the pandemic. Recognizing the importance of health professionals – and, with due emphasis here, of nursing professionals – is a condition for a broad and deep view for humanity to understand health as a complex, dynamic, and plural system.

In this regard, it should be noted that, as a result of the Covid-19 pandemic, challenges related to the shortage of sufficient and qualified labor, as well as insufficiency of materials and infrastructure, were imposed on workers and on health services^(6,7)^.

However, despite this being a reality for a significant part of the health systems - even before the pandemic –, what was perceived was the naturalization of these insufficiencies. Therefore, it should be noted that for complexity, Edgar Morin signals the presence of risks, illusions, and uncertainties as inseparable elements to the existence of humanity. In this premise, it is inferred that man surrounds himself with illusions (scant investment in the health system), drives uncertainty (a health crisis may or may not arrive), and potentiates risks (when a health crisis is installed amid the chaos projected in the health system)^(4)^.

To deal with this human condition, complexity highlights the role of strategies, which, in their turn, do not imply the nullity of risks, uncertainties and illusions, but only help to deal in the best way possible with all these elements^(4)^. Therefore, it is necessary to understand the multidimensionality that structures each complex phenomenon and makes up the objects of intervention, not being different with the Covid-19 pandemic. When this does not happen, the strategies emerge with expressive, linear, and immediatist limitations – the Intensive Care Units (ICU), for example, created in improvised environments, while nursing professionals continue without decent working conditions. Consequently, these workers were overloaded, increasing the chances of illness from the affected physical and mental dimensions^(7)^.

Moreover, the lack of training, the insufficiency of Personal Protective Equipment (PPE), and inconsistent protocols heavily affected the illness process^(8)^. These conditions in the work environment can compromise the professionals’ enthusiasm and satisfaction, and also reflect on the senses, perceptions, and meanings attributed by them to the work of nursing during the Covid-19 pandemic in Brazil, taking into account the economic, political, and social aspects the reality imposed by this pandemic presented to them.

In this setting, reflecting on the meanings of nursing work during the Covid-19 pandemic in Brazil is important, given that these professionals are in the planning and front line of care for users and at different levels of health care. In addition, they represent the largest workforce in the health area and are responsible for direct and uninterrupted care to service users.

Another factor that must be taken into account - when highlighting the importance of understanding the meanings revealed to work, in this context – is the fact that the precariousness of work in the health area has been widely perceived and discussed among service managers, professionals, and users^(9)^. In addition, it mainly concerns the damage that this causes to the health sector, requiring a reflection on how to reverse the problem. This way, managers can develop actions in the Brazilian Public Health System (*SUS*) to guarantee and ensure decent working conditions for professionals, reflecting on the quality of care provided to service users^(10)^.

Furthermore, the discussion about work and its meanings is considered topical and the object of numerous investigations, having in view that work is attributed a fundamental value of the human being and of extreme importance for personal fulfillment. Additionally, it contributes to the progression and development of each citizen’s, nursing professional’s, and health worker’s identity^(11)^.

Therefore, this article grows out of the following guiding question: considering the multidimensionality that structures and affects the work of care nursing in the Covid-19 pandemic, what meanings are connected with this reality? The objective of this study was to reflect on the meanings of the work of Brazilian nursing care in the context of the Covid-19 pandemic.

From this perspective, the reflection presented here is based on the definition of the meaning of work given by Estelle Morin, and on the importance of work organization and the autonomy of nursing professionals and the meanings they attribute to work in a time of pandemic, as Covid-19 in Brazil. In addition, reflecting on the activities carried out during this period will contribute to improving the work process of these professionals in the health crisis.

### Situating Nursing Care Work in the Covid-19 Pandemic

The current pandemic brought to light the precarious conditions of health services in Brazil, with an impact on the workers’ health, especially those of nursing. This fact can be attributed to several situations: lack of adequate working conditions; lack of a clear public policy to face the pandemic; lack of protocols and user flows to regulate and provide more safety to professional performance; accomplishment of exhaustive workload; deficit in training and qualification to face the crisis; and uncertainties arising from the lack of effective treatment to combat the new coronavirus^(12)^.

It is worth highlighting that many of these situations already existed before the pandemic, both in public and private institutions, being more accentuated in private ones, in what regards the type of employment relationship. This relationship has made the precariousness of work more evident due to temporary agreements, object of union movements and class representation of the category. The pandemic situation only aggravated the work process with regard to the conditions and organization of nursing work. This was, unfortunately, a reality experienced by all professionals in the category in the national context^(9,13)^.

In addition, nursing professionals have a hierarchical work system regulated by law, which differentiates those with university and high-school levels. Within this context, a technical and social division is established by the categories, which can give different meanings to the work based on economic, political, and social factors^(14)^. This perception was more evident during the Covid-19 pandemic in Brazil, with the possibility of a double and even triple shift, further increasing the work overload, while demonstrating the precariousness of nursing work, evidenced, above all, by the low remuneration^(15)^.

Therefore, the Covid-19 pandemic has imposed a significant challenge to nursing professionals, given their leading role in fighting and coping with this serious health crisis, highlighting the need for autonomous and safe action and emphasizing its importance in dealing with the pandemic^(16)^.

The Covid-19 pandemic has imposed sanitary challenges on health workers, underlining how serious the current crisis is, especially when the growing social inequality is observed. Workers and the population underwent significant impacts related to their health status, a fact that justifies considering the contribution of nursing professionals in a projection of public interest for the development of nations. In this setting, one can infer the reiteration of their social role, in defense of life, besides allowing universal access to health, required to face social inequities, which tend to increase in the post-pandemic period^(17)^.

This way, the work developed by nursing professionals, not only through direct patient care, but also in the management of strategies for the organization of health services, became even more evident with the health systems facing the pandemic. However, the movement to value nursing as a condition for health systems around the world not to collapse, amidst the current epidemiological challenges and those foreseen for the near future, was signaled, before the Covid-19 pandemic, as from the campaign *Nursing Now*, released in 2018^(18)^.

This global campaign aimed to draw the attention of civil society – and government leaders – to the due appreciation and empowerment of Nursing professionals from all over the planet. Therefore, among the documents motivating its organization, there is the Triple Impact Report, which signaled to the British Parliament the importance of investments in nursing, without which it would not be possible to achieve significant impacts on health, in the reduction of inequalities related to gender, and in the economy^(19,20)^.

As it could be globally seen, in 2020, while the bicentennial of Florence Nightingale and the International Year of Nurses and Midwives were celebrated, the challenges of health systems increased and, therefore, the need for investments in nursing to face the pandemic that afflicted contemporary humanity. Despite the foregoing, in Brazil, the precarious work in health was sometimes camouflaged in momentary applause from people in their windows, soon suppressed due to the increasing numbers of morbidity and mortality of health professionals, especially from the nursing category, during the course of that year and the following one.

Although the course of the Covid-19 pandemic took place from a tangle of historical contexts for nursing, it is important that these professionals recognize their central role as agents of care and know the value of the nursing profession. This reality may lead to mobilization for the due investment in qualification and training in a continuous way and with the full exercise of citizenship, social awareness, and the development of strategies capable of stimulating the appreciation and professional recognition of nursing^(21)^.

In addition, according to data from the Observatory of the Federal Nursing Council (*Cofen*), extracted in early July 2022, 64,148 nursing professionals – nurses, technicians and assistants – were infected with the new coronavirus and 872 died as a result of the disease, equivalent to 2.31% lethality^(22)^. It is important to note that the numbers do not represent the totality of cases and deaths, in view of possible underreporting.

The increase in cases of illnesses and deaths of nursing professionals became more visible in the media when the Observatory was created by *Cofen*. The fear of getting Covid-19 when taking care of the sick user, the fear of transmitting it to a family member, added to the work overload and the lack of PPE, led many professionals to develop mental disorders – such as panic disorder and depression -, to resign from their job, or simply leave it^(23)^.

With this scenario, it is possible to identify feelings that arise in this moment of challenge imposed by the Covid-19 pandemic – fear of the unknown, anguish, and concern, for example, as well as to note the presence of anger due to the impotence of not being able to go beyond, among other problems, that compromise this professional’s mental health^(1,24)^. The feelings mentioned are perceived due to uncertainty or doubt about the disease prognosis and the social isolation these workers impose themselves – to protect their families –, besides experiencing financial and social conditions that do not allow them to give up or leave their job^(1)^.

Moreover, challenges such as overwork and lack of adequate staffing were added with the growth in the number of absenteeism, illness and deaths, increasing the burden, devaluation, and precariousness of the service, compromising the quality of care. This fact shows how important is political engagement for an effective search for appreciation of their work. Benefiting from the visibility given by the pandemic, nursing professionals have to make it clear and demand permanent qualification, decent wages, adequate working environments and conditions, and fair workload^(25)^.

### Unveiling Meanings of the Work of Nursing Care in Times of the Covid-19 Pandemic

For some time, scholars have sought, through different approaches and methodologies, to understand the meanings that people assign to work in different places, times and social, economic and political contexts. However, two possibilities overlapped: admitting that work occupies a place of neutrality; and, on the other hand, that it also has a condition of centrality in the perception of workers, influencing these individuals’ personal and social identity^(26)^.

Estelle Morin and collaborators^(26)^ state that work only makes sense if it is intensified and capable of producing profit in a capitalist and neoliberal logic, while Ricardo Antunes defends that the meaning of work needs to go beyond capital. There is no clear definition or consensus on the concept of the expression meanings of work, although there is an approximation in what concerns comprehending it as a convergence of individual desires and the achievements accomplished. Thus, it is important to reflect on the meanings of work starting from the universal and individual concepts, considering the notion of salaried work from a liberating and citizenship perspective^(27)^.

Also according to Estelle Morin and collaborators^(26)^, work is fundamental in people’s lives and is associated with issues related to each individual’s sustenance and subsistence. Furthermore, the meanings of work are also related to values such as the variety of professional activities, the possibility of establishing teaching, learning and qualification, as well as professional autonomy, stability, and financial safety.

The theoretical-methodological approach of this article is supported by the definitions of meanings of work established by Estelle Morin^(28)^, which are based on the existentialist approach, which considers that people’s actions need to have meaning, being an affective framework that is established based on three elements: (a) meaning, which concerns the individual, how he/she represents and attributes value to his/her job; (b) guidance, which refers to what the worker looks for in his/her work; and, finally, (c) coherence, that is, the expected harmony between their environment and their relationship with work. On this basis, meanings are influenced since the moment workers start to have positive attitudes towards the work organization.

In addition, and still based on the assumptions established by Estelle Morin^(28)^, the work only makes sense when the professional has more knowledge about it, being able to make its organization reach effectiveness. Estelle also highlights that the meaning attributed by professionals to work is related to autonomy, recognition, professional progression and development.

With this, the following reflections intend to describe the meanings of nursing work during the Covid-19 pandemic in Brazil, based on the theoretical assumption defended by Estelle Morin^(26,28)^, aiming to understand the meanings of work assigned by nursing professionals in peculiar situations, at the time of the pandemic. Furthermore, that it promotes reflection on the need to think about another form of work, in a critical, reflective and citizenship perspective capable of resignifying their professional and organizational activity with political, economic, social, and autonomous thinking.

Estelle Morin^(26,28)^ assigns meanings to work based on three dimensions: individual, social, and organizational. The individual concerns the meaning that the individual attributes to work; social refers to a person’s ability to contribute to society; and organizational refers to the meaning attributed to work based on its usefulness and recognition, that is, it addresses issues of interpersonal relationships and the exercise of citizenship.

Thus, the contemplations on the impacts of the performance of nursing professionals during the Covid-19 pandemic in Brazil are related to the economic issue, linked to the need for survival as a meaning of work. Also highlighted are the social values related to the possibility of performing different tasks, exercising the profession with autonomy, recognition and safety, which are extremely important elements for the meaning of work to exist and be significant^(26)^.

The work that makes sense is the one that brings meaning and presents a purpose, that is, it is the one that has an objective^(28)^. During the Covid-19 pandemic, the incorporation of this meaning by the nursing workers became even more important, given that the society recognized their relevance during the fight against the pandemic, and this allowed the discussion about the social, political, and economic recognition of this worker.

In this context, it is possible to understand that, for nursing professionals, the meaning of work corresponds to empowerment and the possibility of ascending economically through an income consistent with their work process^(14)^. In this regard, it is important to highlight that the desire for economic growth contrasts with low remuneration, which can discourage and give a negative meaning to the work they do.

Work is understood as an important value by society and by all professionals, and is directly related to their motivation – in addition to productivity, in a capitalist perspective –, being able to provide professional satisfaction^(28)^. Nursing professionals represent the largest workforce working in the health area, and this gives them the status of producer of consumer goods – nursing care –, adding value and social meaning. With this representation, it is expected that the professional reaches the individual dimension, mentioned by Estelle Morin, translated into the representation that the work has for the individual and the value it is attributed.

In addition, with the advent of the Covid-19 pandemic, the social value assigned to nursing received greater prominence, going beyond the convictions of these professionals – experiences lived in times of health crisis, which reflect on working and living conditions. It is worth noting that social values result from economic values; however, it is in this pandemic moment that the contradictions between the great responsibility required from these professionals and the difficulties related to the work environment and labor issues are evident. There is great repercussion in the media about the work of nursing professionals from the social criticism evidenced by the pandemic, providing greater visibility and encouraging more discussion. All this, however, without causing a political impact that allows for better working conditions, non-precarious employment relationships, better remuneration, and fair weekly working hours^(25)^.

Nevertheless, it can still be said that nursing has not lost sight of what Estelle Morin^(28)^ states regarding the social dimension. In other words, it provides subsidies to the meaning given to work when, despite all the problems triggered during the Covid-19 pandemic, this group of workers managed to accomplish the great social contribution made with their work.

In this regard, nursing professionals have gained greater visibility and, in a way, professional and social recognition, given that they have been working on the front line against the pandemic at all levels of health care, while the real working conditions of these professionals were evidenced. Notwithstanding this recognition contributes to the organizational dimension^(28)^ – based on the usefulness and recognition of the work performed by the nursing professional, as well as on the exercise of citizenship –, its main claims concern the working day with the establishment of a workload of 30 hours per week, and a wage floor through a Federal Law.

It should be noted that, in May 2022, the Chamber of Representatives approved the Bill (PL) no. 2.564/2020 that deals with the national wage floor for nurses, nursing technicians, nursing assistants, and midwives. According to the aforementioned bill, the nurse shall receive at least R$ 4,750.00; the nursing technician, 70% of that; the nursing assistant and midwife, 50% of this wage floor. To ensure the regulation and constitutionality of the aforementioned bill, the Proposed Amendment to the Constitution (PEC) no. 11/2020 was approved, in two shifts, and promulgated in the Federal Senate and Chamber of Representatives, in June and July, 2022. On August 4, 2022, the President of the Republic sanctions Law No. 14.434, which establishes the national nursing wage floor, with a veto to the annual correction of the floor, by the National Consumer Price Index (INPC). However, the professional category has historically and politically mobilized at the Federal Senate to overturn the veto.

In a way, this history represents a great achievement to the discussions about the wage floor of the category, which for years has been the object of professional struggle. Such a situation is often found in the professionals’ discourse, leading them to reflect on whether or not to maintain the profession. Now, it is up to the supervision agencies of professional practice to seek compliance with the Law and guarantee the rights of nursing professionals, since large medical centers, particularly those of a private nature, have been mobilized against its application.

Such facts are reflected in the way in which these professionals attribute meaning to the work they perform, because, despite great recognition, they are still subjected to long working hours, low remuneration, and the absence of public policies for professional valuation – such as the establishment of a minimum wage and definition of working hours.

Also according to Estelle Morin^(26,28)^, when describing the meanings of work for young Brazilian executives, meaningless work is the work that does not allow the professional to grow intellectually, financially, and to acquire knowledge, as well as being unable to explore the worker’s potential, his/her development and professional progression.

Henceforth, when reflecting on meaningless work and bringing this reflection to the field of nursing professionals, it is worth noting that hiring, the work regime, and the remuneration attributed reflect on a negative or meaningless perception, when faced with a health service that does not recognize their work from a social, political, and economic point of view.

Due to the crisis caused by the Covid-19 pandemic in Brazil, the job market – which was already in decline, mainly after the Labor Reform, enacted in 2017 through its own legislation, which changed the forms of hiring - became even more difficult in what regards guarantees and labor safety. In this respect, all classes of workers and various economic sectors were profoundly affected in different ways. However, it is worth noting that there is social inequality when we reflect on the economic difficulties in different social classes and in different regions of Brazil^(29)^.

In addition, the work that is meaningless refers to work without motivation, only carried out for the salary return and without identification, as well as that without perspectives and without results, repetitive, with no autonomy, and precarious. In this moment of precariousness of work, through agreements with no safety or stability for the worker, conditions are imposed that give the direction to the meaning of work. Thus, the nursing work that is meaningless during the Covid-19 pandemic in Brazil is the one without individual and collective perspectives and objectives, without intellectualization, and that does not recognize the important social contribution and the enormous capacity to organize the service and the work process.

## FINAL CONSIDERATIONS

The meanings of work attributed by nursing professionals reflect the social, political, and economic moment in Brazil today. Nursing professionals have suffered greatly with the advent of the Covid-19 pandemic, given that they have an exhausting workday, receive low remuneration, and show negligible awareness and political mobilization.

In nursing, there has not been, for many years, an articulation capable of having its political demands met, such as the establishment of a 30-hour workweek and the approval of the national salary floor, which has been in progress for more than 20 years, and only recently has it gained visibility given the social role of nursing in the face of Covid-19, aiming to give more dignity to these workers.

Thus, the work with meaning for nursing professionals, during the Covid-19 pandemic, is related to recognition through better salaries, attributing value to these workers’ workforce, that is, relating to the need for sustenance. On the other hand, meaningless work is one that does not allow intellectual, cognitive, and especially financial advances, making professional growth and development unfeasible.
